# Beneath the surface: a case report of metastatic iridociliary ring melanoma treated with pembrolizumab

**DOI:** 10.5935/0004-2749.20230046

**Published:** 2023

**Authors:** Cinthya Parra-Bernal, Jorge A. Aguilera-Partida, Lauren A. Dalvin, Abelardo A. Rodríguez-Reyes, David Ancona-Lezama

**Affiliations:** 1 Ocular Oncology Service, Institute of Ophthalmology and Visual Sciences, Tecnologico de Monterrey, Monterrey, Mexico; 2 Uveitis and Ocular Inflammation Service, Clínica Oftalmológica Santa Lucía, Guadalajara, Jalisco, Mexico; 3 Department of Ophthalmology, Mayo Clinic, Rochester, Minnesota, United States; 4 Ophthalmic Pathology Service, Asociación Para Evitar La Ceguera en México, Hospital Dr. Luis Sanchez Bulnes, Mexico City Mexico

**Keywords:** Uveal neoplasms/complication, Melanoma, Iris neoplasm/secondary, Ciliary body, Antibody, monoclonal, humanized, Immune checkpoint inhibitor, Humans, Case report, Neoplasia uveal/complicação, Melanoma, Neo-plasia da íris/secundário, Corpo ciliar, Anticorpo monoclonal humanizado, Inibidor de checkpoint imunológico, Humanos, Relato de caso

## Abstract

Iridociliary ring melanoma is an uncommon type of uveal melanoma. Clinical manifestation varies from asymptomatic cases to masquerade syndromes mimicking refractory glaucoma. Treatment options include radiotherapy and enucleation. Management of metastatic uveal melanoma remains discouraging. Novel therapies using immune checkpoint inhibitors are currently under study. We present a case of a 54-year-old Hispanic woman with progressive vision loss due to metastatic ring melanoma with anterior chamber seeding treated with pembrolizumab.

## Abstract (pt)

O melanoma iridociliar em anel é um tipo incomum de melanoma uveal. As manifestações clínicas variam desde casos assintomáticos até síndromes mascaradas que mimetizam um glaucoma refratário. As opções de tratamento incluem radioterapia e enucleação. O manejo do melanoma uveal metastático continua desanimador. Novas terapias usando inibidores de *checkpoint* imunológico estão atualmente em estudo. Apresentamos o caso de uma mulher hispânica de 54 anos com perda progressiva da visão por um melanoma metastático em anel, com semeadura de câmara anterior, tratada com pembrolizumabe.

## INTRODUCTION

Annular or ring melanoma has been described as a diffuse variant of uveal melanoma that extends circumferentially^(1)^. This unusual growth pattern can appear in any structure pertaining to the uveal tract, including the iris, ciliary body, and choroid^(2)^. Currently, the true incidence of iridociliary melanoma is unknown. Additionally, clinical manifestations and ocular findings may vary widely, ranging from asymptomatic to complex cases mimicking refractory pigmentary or neovascular glaucoma. As a result, definitive diagnosis and treatment are often delayed.

We hereby describe our clinical approach in a patient with ring melanoma simultaneously involving the iris and ciliary body that developed anterior chamber seeding over the course of the disease. Furthermore, to the best of our knowledge, this is the first reported case of metastatic ring melanoma in the Hispanic population treated with an immune checkpoint inhibitor (ICI).

## CASE REPORT

A 58-year-old Hispanic woman with no past medical history of systemic disease was referred to our clinic complaining of a red, painful left eye (OS) and progressive vision loss. A trabeculectomy of OS was performed elsewhere due to refractory ocular hypertension two months before the admission.

**Figure 1:**
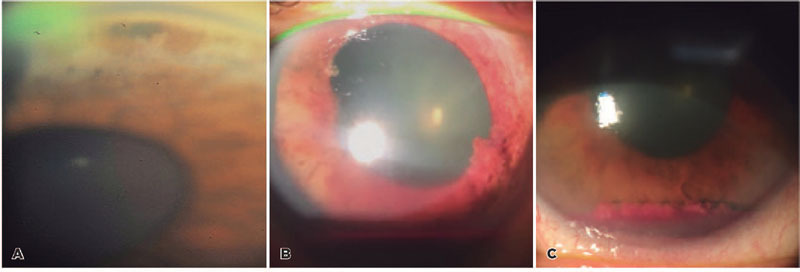
Slit-lamp findings. On gonioscopy, (A) distortion of the iridocorneal angle and hyper- and hypopigmentary changes were identified on the iris surface. Two months later, anterior segment examination revealed (B) hyphema and a newly developed exophytic mass in the inferior pupillary margin. Prior to fine-needle aspiration biopsy, (C) the previously described mass appeared free-floating in the anterior chamber.

Best-corrected visual acuity was 20/40 in her right eye (OD) and 20/50 in OS. Intraocular pressure (IOP) was 12 mm Hg in OD and 5 mm Hg in OS. Slit-lamp exam of OD was unremarkable. OS was positive for sectoral iris neovascularization and peripheral pigmentary changes. Gonioscopy revealed a distortion of the iridocorneal anatomy, and a 1 mm x 1 mm pigmented lesion surrounded by white velvety infiltration (Figure 1A). Ultrasound biomicroscopy (UBM) was suggested as the next step, but the patient was lost to follow-up.

**Figure 2:**
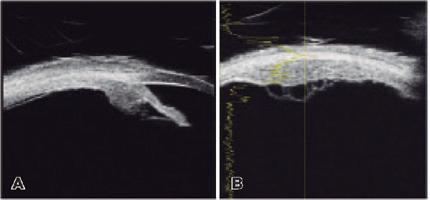
Anterior segment ultrasound biomicroscopy. (A) Ultrasound biomicroscopy of the left eye revealed circumferential infiltration of the ciliary body (arrow) with (B) internal cystic spaces.

Two months later, the patient returned complaining of new-onset blurry vision in OS. Anterior segment examination revealed a grade-1 hyphema and a new exophytic mass on the inferior pupillary margin with a similar color to that of the iris (Figure 1B). IOP in OS was 21 mm Hg. Anterior segment UBM of OS revealed a hollow mass that infiltrated the ciliary body in an annular pattern (Figure 2A and 2B). A transscleral biopsy was planned to rule out uveal melanoma. Three days later, the iris mass in OS was floating freely in the anterior chamber with an associated rise in IOP due to profuse anterior chamber seeding (Figure 1C).

**Figure 3:**
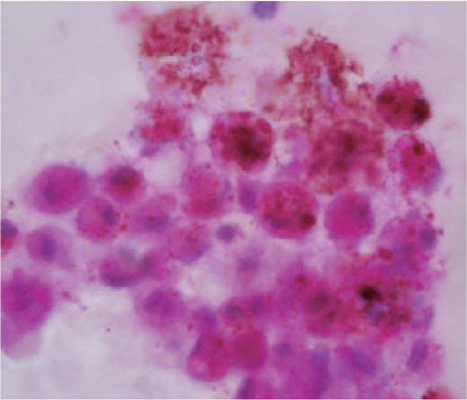
Cytology. Epithelioid cells with intracytoplasmic melanin, nuclear atypia, and few mitotic figures, intermingled with melanophages and red blood cells (H&E, original magnification 100x).

Anterior chamber fine-needle aspiration biopsy (FNAB) was performed, and cytology was consistent with melanoma of the iris and ciliary body (Figure 3). Systemic workup revealed lung and brain metastases by computed tomography. Biopsy of metastatic lesions was not performed. The patient received brain radiotherapy sessions and four pembrolizumab (Keytruda®, Merck Sharp & Dohme Corp, Kenilworth, N.J., U.S.A.) intravenous cycles. The appearance of multiple small nodules on the iris surface and uncontrolled high IOP suggested ocular progression despite treatment. Unfortunately, vision worsened during follow-up, ultimately reaching hand-motion vision. The patient died 40 days after the fourth cycle due to metastatic complications.

## DISCUSSION

Ciliary body ring melanoma accounts for only 0.3% of all uveal melanomas^(3)^. It is often overlooked on routine ophthalmic examination due to its occult position during early stages. As the tumor grows in size, it can compromise other intraocular structures, causing intraocular inflammation, elevated IOP, shallow anterior chamber, lens displacement, and cataract formation^(3)^.

Interestingly, up to 13% of ciliary body ring melanomas are initially approached as glaucoma before suspecting malignancy^(3,4)^. Proposed mechanisms for secondary glaucoma in ciliary body melanoma include seeding to or direct invasion of the anterior chamber angle and angle-closure glaucoma caused by synechiae, anterior displacement of the iris-lens diaphragm, or neovascularization^(5)^. Therefore, uveal melanoma should always be considered in the setting of a refractory unilateral pigmentary or neovascular glaucoma, such as in this case.

Non-invasive diagnostic techniques, including transillumination, comparative gonioscopy, B-mode ultrasound, and UBM are encouraged. In this case, dense pigmentation on gonioscopy, intrinsic hollowness on ultrasound, and circular involvement of the ciliary body were suggestive of ring melanoma. FNAB has been proven as a safe tool for the diagnosis of iris tumors^(6)^. Based on the iris tumoral mass formation and anterior chamber seeding in our patient, FNAB was chosen to confirm the diagnosis of iridociliary ring melanoma.

There is no well-established therapeutic algorithm for ring melanoma. Gündüz et al. reported a local control rate of 92% at five years using plaque radiotherapy in patients diagnosed with uveal melanoma with predominant ciliary body involvement^(7)^. Enucleation or exen-teration has been advised in large ciliary body melanomas, extraocular extension, or refractory glaucoma^(3)^.

The extraocular spread of uveal melanoma occurs mostly through aqueous drainage channels, particularly in those tumors involving the ciliary body or anterior chamber angle^(8)^. Delayed diagnosis and glaucoma filtration procedures might also increase the risk of extra-ocular extension^(9)^. These characteristics confer ciliary body ring melanoma a poorer prognosis compared to choroidal melanoma and non-diffuse tumor configurations^(3)^. As such, periodic liver function tests and systemic imaging are warranted.

Different chemotherapeutic regimens and novel immunotherapy options are currently under study in the setting of metastatic uveal melanoma (mUM). Anti-programmed cell death receptor 1 (PD-1)/ programmed death ligand 1 (PD-L1) antibodies (pembrolizumab and nivolumab) are ICIs that block the interaction between PD-1 and PD-L1, promoting a T-cell response against tumor cells^(10)^. Alongside anti-cytotoxic T-lymphocyte-asso ciated antigen 4 (CTLA-4; ipilimumab), anti-PD-1/ PD-L1 antibodies are currently approved for metastatic melanoma^(10)^. So far, studies have yielded lower responses to ICIs in mUM compared to metastatic cutaneous melanoma (mCM)^(11)^. Klemen et al. recently reported a median overall survival (OS) of 12 months and 22% of 5-year survival after treatment with ICI in patients with mUM whereas patients with mCM experienced an OS of 45 months and 46% of 5-year survival^(12)^. Similarly, a multicenter retrospective study demonstrated a progression-free survival (PFS) of 2.6 months, OS of 7.7 months, and an overall response rate (ORR) of 3.6% in patients with mUM who received treatment with pembrolizumab or nivolumab, regardless of previous ipilimumab usage^(13)^. Few data have been obtained from treatment-naïve patients with mUM. A small cohort of 17 patients who received pembrolizumab as first-line therapy for mUM showed PFS of 3.8 months and ORR of 11.7%^(14)^. PFS was slightly increased in patients with an interval longer than 5 years from the diagnosis of the primary tumor to metastases and in those with extrahepatic metastases^(14)^. Differences in oncogenic mutations, PD1/PD-L1 patterns of expression, and a number of tumor-infiltrating lymphocytes have been proposed as key factors for response variability toward ICI in uveal melanoma^(15)^. In this case, pembrolizumab proved to have limited efficacy in local and metastatic control.

Ring melanoma is an uncommon type of uveal melanoma. Its inaccessibility on slit-lamp examination poses a special challenge for diagnosis and demands the highest awareness and preparedness from a multi-disciplinary approach. The efficacy of ICIs for ring uveal melanoma needs to be further researched to determine if there is any useful role in local and systemic disease control. Poor prognosis and low survival rates urge the need for further studies and effective treatment for primary malignancy and metastatic disease.
